# Aposymbiosis of a *Burkholderiaceae*-Related Endobacterium Impacts on Sexual Reproduction of Its Fungal Host

**DOI:** 10.1264/jsme2.ME19167

**Published:** 2020-04-15

**Authors:** Yusuke Takashima, Yousuke Degawa, Tomoyasu Nishizawa, Hiroyuki Ohta, Kazuhiko Narisawa

**Affiliations:** 1 United Graduate School of Agricultural Science, Tokyo University of Agriculture and Technology, 3–5–8 Saiwai-cho, Fuchu-shi, Tokyo 183–8509, Japan; 2 Ibaraki University College of Agriculture, 3–21–1 Chuo, Ami-machi, Ibaraki, 300–0393, Japan; 3 Sugadaira Research Station Mountain Science Center, University of Tsukuba, 1278–294, Sugadaira, Nagano 386–2204, Japan

**Keywords:** *Burkholderiaceae*-related endobacteria, homothallism, *Mortierella*, zygospore

## Abstract

Bacterial endosymbionts inhabit diverse fungal lineages. Although the number of studies on bacteria is increasing, the mechanisms by which bacteria affect their fungal hosts remain unclear. We herein examined the homothallic isolate, *Mortierella sugadairana* YTM39, harboring a *Burkholderiaceae*-related endobacterium, which did not produce sexual spores. We successfully eliminated the bacterium from fungal isolates using ciprofloxacin treatment and asexual spore isolation for germinated asexual spores. Sexual spore formation by the fungus was restored by eliminating the bacterium from isolates. These results indicate that sexual reproduction by the fungus was inhibited by the bacterium. This is the first study on the sexual spore infertility of fungal hosts by endofungal bacteria.

Recent studies using molecular techniques revealed that bacterial endosymbionts inhabit early diverging fungal lineages, such as Mucoromycota (Spatafora *et al.*, 2016; Bonfante and Desirò, 2017). These bacterial endosymbionts were known as endofungal or endohyphal bacteria and mostly related to the family *Burkholderiaceae*, and, thus, were termed *Burkholderia*-related endobacteria (Bonfante and Desirò, 2017) or *Burkholderiaceae*-related endobacteria (BRE) (Takashima *et al.*, 2018b). BRE were generally found in hyphae and asexual spores (Lumini *et al.*, 2007; Sato *et al.*, 2010; Ohshima *et al.*, 2016; Mondo *et al.*, 2017; Takashima *et al.*, 2018a) as well as in sexual spores (Mondo *et al.*, 2017). The host nutrient dependence of BRE due to genome reductions was suggested by genomic studies (Ghignone *et al.*, 2012; Fujimura *et al.*, 2014; Uehling *et al.*, 2017; Sharmin *et al.*, 2018). However, limited information is currently available on the effects of BRE on their fungal hosts.

The elimination of BRE from their fungal hosts previously revealed several effects on their hosts. BRE were found to alter gene expression (Lastovetsky *et al.*, 2016; Salvioli *et al.*, 2016; Uehling *et al.*, 2017), metabolism (Salvioli, *et al.*, 2010; Lastovetsky *et al.*, 2016; Li *et al.*, 2017), oxidative stress responses (Salvioli *et al.*, 2016; Vannini *et al.*, 2016; Venice *et al.*, 2017), and chemotaxis (Lumini *et al.*, 2007) in hosts. In contrast, only a few studies described the morphological alternations induced by BRE. The wall thickness of the chlamydospores of *Gigaspora margarita* was greater (Lumini *et al.*, 2007), the sporangiospores of *Rhizopus microsporus* were not produced (Partida-Martinez *et al.*, 2007; Lackner *et al.*, 2011), and the aerial mycelia of *Mortierella elongata* were well developed (Uehling *et al.*, 2017) in the absence of each BRE. In zygospore formation by *R. microsporus*, the number of zygospores produced in mating was lower for *Burkholderia*-free strains than for *Burkholderia*-harboring strains (Mondo *et al.*, 2017). BRE are currently considered to be beneficial and non-harmful endosymbionts for their hosts.

*Mortierella* spp. produce sporangiospores and zygospores as asexual and sexual spores, respectively, in their life cycle for dispersal, dormancy, and recombination at the sexual stage. We recently obtained three fungal isolates, YTM39, YTM128, and SUT-174, from cool regions in Hokkaido and Nagano, Japan (Table 1). These isolates were described as *Mortierella sugadairana*, which form zygospores in a homothallic manner and are related to the first heterothallic species described in the genus (Takashima *et al.*, 2018a). Among them, one isolate, *M. sugadairana* YTM39, did not form zygospores under zygospore-inducing conditions, whereas the other two did. The isolate, *M. sugadairana* YTM39, was different from the other two isolates because it harbored a BRE, which was phylogenetically clustered within MorBRE group C assigned by Takashima *et al.* (2018b). Based on the differences observed in zygospore formation by the isolate, *M. sugadairana* YTM39 and the two other isolates as well as the presence of the bacterium, we hypothesized that the bacterium affects zygospore formation by this isolate. In order to prove this hypothesis, we attempted to eliminate the bacterium from *M. sugadairana* YTM39 and observed zygospore formation by BRE-harboring and BRE-free clonal lines obtained from the original line. The results obtained showed that zygospore formation was restored in the BRE-free clonal lines of the isolate. The present study is the first to show sexual spore infertility by a fungal host in the presence of BRE.

## Materials and Methods

### Elimination of BRE from the fungal host

In order to eliminate BRE from the fungal host, we employed antibiotic treatments, such as ciprofloxacin, which was previously shown to be effective against BRE associated with *R. microsporus* (Partida-Martinez *et al.*, 2007). To assess the ciprofloxacin treatment, sporangiospores of the original isolate *M. sugadairana* YTM39 (wild-type; BRE-harboring [B+]) and two clonal lines (clonal line names including “s” and “mc” hereafter indicate clonal lines obtained by single-sporangiospore isolation and the ciprofloxacin treatment, respectively) generated by the following single-sporangiospore isolation, *M. sugadairana* YTM39s3 (B+) and YTM39s4 (B+), were used. Small agar pieces containing several germinated sporangiospores were added to separate 1.5-mL tubes for each clonal line. Four, ten, and ten small agar pieces were obtained from *M. sugadairana* YTM39 (B+), YTM39s3 (B+), and YTM39s4 (B+), respectively. To eliminate endofungal bacteria, 500‍ ‍μL of 50‍ ‍μg mL^–1^ ciprofloxacin hydrochloride (Wako Pure Chemical) solution was added to the tubes, which were then incubated at room temperature (*ca.* 23°C) for 24 h. After the incubation, agar pieces were disrupted in the solution by pipetting, and all of the solution was inoculated onto fresh _LC_A (Miura agar) medium (0.2‍ ‍g yeast extract [Difco], 1‍ ‍g glucose [Wako], 2‍ ‍g NaNO_3_ [Wako], 1‍ ‍g KH_2_PO_4_ [Wako], 0.2‍ ‍g KCl [Wako], 0.2‍ ‍g MgSO_4_·7H_2_O [Wako], and 15‍ ‍g Bacto agar [Difco] in 1 L distilled water) (Miura and Kudo, 1970). After mycelia appeared, a small agar piece containing a few hyphal tips was cut and inoculated onto fresh _LC_A medium to establish ciprofloxacin-treated clonal lines that were potentially free from the bacterium.

### Single-sporangiospore isolation

Although ciprofloxacin is an antibacterial agent, a previous study reported that it functioned as an abiotic stress factor in some fungi (Yuan *et al.*, 2011). To avoid placing any abiotic stress on the fungal hosts by the addition of chemicals, single-sporangiospore isolation was also conducted to obtain BRE-free clonal lines of the fungal host. In single-sporangiospore isolation, sporangiospores of *M. sugadairana* YTM39 (B+) were spread onto _LC_A medium. Twelve and thirty-four germinated sporangiospores of the original *M. sugadairana* YTM39 were obtained from two different plates. These germinated sporangiospores were separately inoculated onto fresh _LC_A medium as the first single-sporangiospore isolation. This procedure was repeated with every newly obtained and selected BRE-harboring clonal lines for the third successive sporangiospore generations. The first clonal lines *M. sugadairana* YTM39s3 (B+) and YTM39s4 (B+), the second clonal line *M. sugadairana* YTM39s3s1 (B+), and the third clonal line *M. sugadairana* YTM39s3s1s6 (B+) obtained by the serial single-sporangiospore isolation were used and ten, ten, seven, and sixteen clonal lines were obtained, respectively. After every single-sporangiospore isolation, the presence of the bacterium was checked using diagnostic PCR.

### Diagnostic PCR

The presence of BRE in each clonal line was checked by diagnostic PCR. Template DNA was extracted from the six- to twelve-day-old mycelia of each clonal line incubated on a sterilized cellophane sheet placed on 1/2 CMMY agar (8.5‍ ‍g corn meal agar [Difco], 10‍ ‍g malt extract [Difco], 1‍ ‍g yeast extract [Difco], and 7.5‍ ‍g Bacto agar [Difco] in 1 L distilled water) using Prepman^TM^ Ultra sample reagent (Applied Biosystems) in accordance with Sato *et al.* (2010). Diagnostic PCR was performed using the bacterial universal primers 10F and 1541R in accordance with Takashima *et al.* (2018b). After PCR, amplification of the 16S rRNA gene was checked by agarose gel electrophoresis.

### Fluorescence *in situ* hybridization (FISH)

In order to confirm the elimination of the bacterium by single-sporangiospore isolation, sporangiospores formed by the representative clonal lines, *M. sugadairana* YTM39s3 (B+) and YTM39s14 (BRE-free [B–]), generated by single-sporangiospore isolation were subjected to FISH. Young germlings of the sporangiospores of each clonal line immobilized by 2% low melting point agar were transferred to a 1.5-mL tube. FISH was performed using agar pieces containing sporangiospores in accordance with Takashima *et al.* (2018b).

### Mycelial growth of BRE-harboring and BRE-free clonal lines

To examine the effects of the presence/absence of BRE and the ciprofloxacin treatment on mycelial growth by the fungal host, mycelial growth by representative BRE-harboring and BRE-free clonal lines of the fungus was investigated in accordance with Takashima *et al.* (2018a). Differences among the means of mycelial growth (mm d^–1^) of each clonal line were statistically evaluated with Tukey’s honestly significant difference (HSD) test (Significance level defined as *P*<0.01) using the R package “multcomp” in R version 3.3.1 (https://www.r-project.org/).

### Zygospore induction

**Fig. 1.**
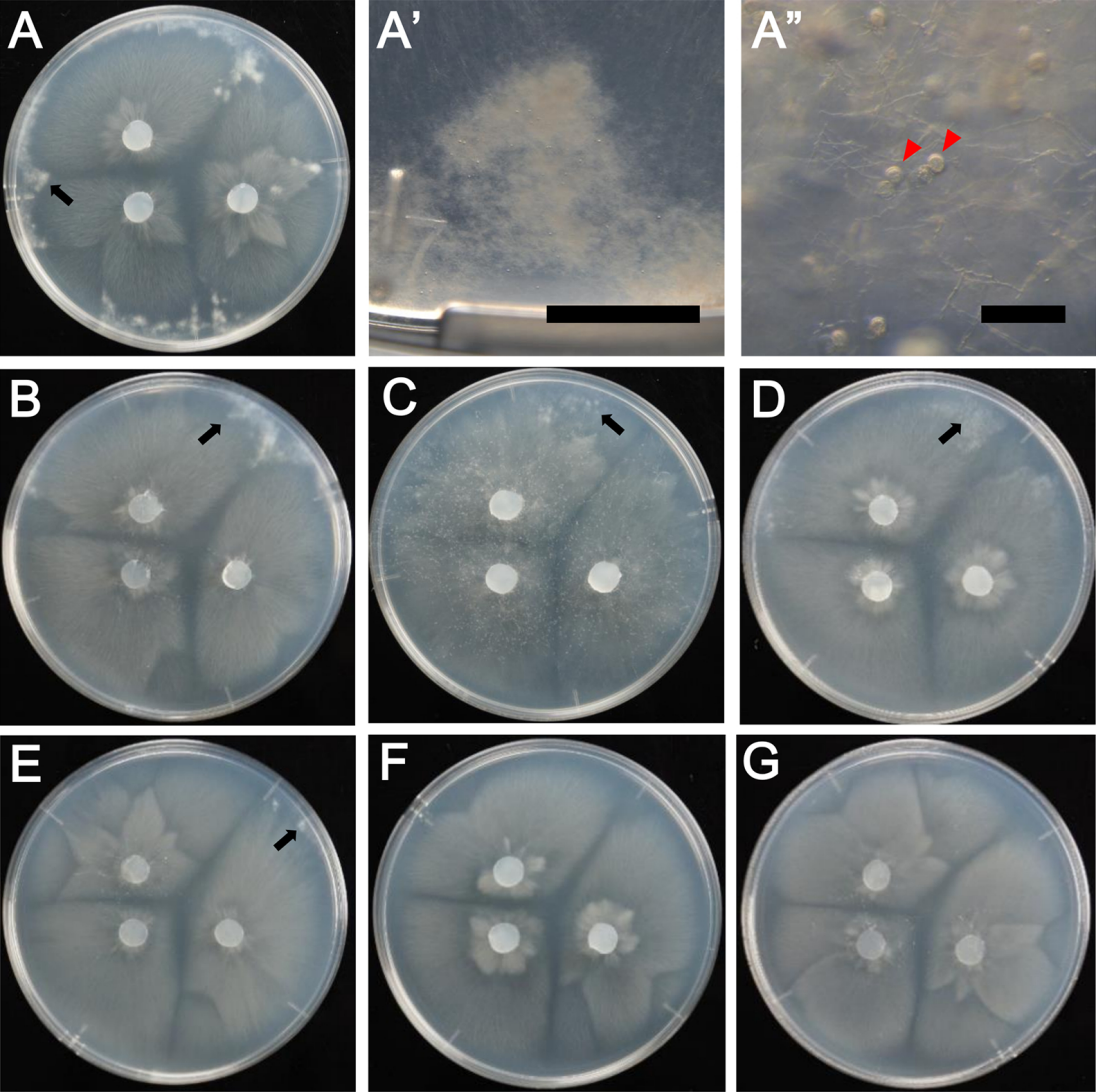
Appearance of colonies on _LC_A incubated for 3‍ ‍weeks at 18°C for homothallic zygospore induction using clonal lines of *Mortierella sugadairana* (A, A’, A”: YTM39s9; B: YTM39s3_mc1; C: YTM39s12; D: SUT-174s1; E: YTM128s1; F: YTM39s3; G: YTM39s3_s1) A, B, C, D, E: Hyphal masses appeared at the edge of 90-mm Petri dishes (Arrows). F, G: Hyphal masses did not form. A’: Magnified images of the section indicated by arrows. A”: Zygospores formed in hyphal masses indicated by arrowheads. Scale bars: A’ 5‍ ‍mm; A” 200 μm.

To induce zygospores, six and sixteen clonal lines of *M. sugadairana* YTM39 obtained by the ciprofloxacin treatment and single-sporangiospore isolation, respectively, were used (Table 2). Among them, 10 out of the 16 clonal lines generated by single-sporangiospore isolation harbored BRE (B+) and the other twelve clonal lines were BRE-free (B–) (Table 2). Regarding the zygospore induction of isolates of *M. sugadairana* YTM128 and SUT-174, single-sporangiospore isolation was also conducted in advance. These clonal lines of *M. sugadairana* YTM39, YTM128, and SUT-174 were incubated on _LC_A agar medium at 18°C for five to seven days prior to zygospore induction. Three inoculation discs were cut from incubated mycelia using an autoclave-sterilized plastic straw (8‍ ‍mm in diameter) instead of a cork borer, and were then placed onto _LC_A medium and hemp seed agar (HSA) (100‍ ‍mL of hemp seed extract prepared by autoclaving 10‍ ‍g of hemp seeds in 100‍ ‍mL of distilled water and 15‍ ‍g Bacto agar [Difco] in 1 L of distilled water), as shown in Fig. 1, with duplicates for each medium, and incubated at 18°C in the dark for three weeks, which are the conditions used in the previous study (Takashima *et al.*, 2018a).

## Results and Discussion

### Elimination of BRE from *M. sugadairana*

**Fig. 2.**
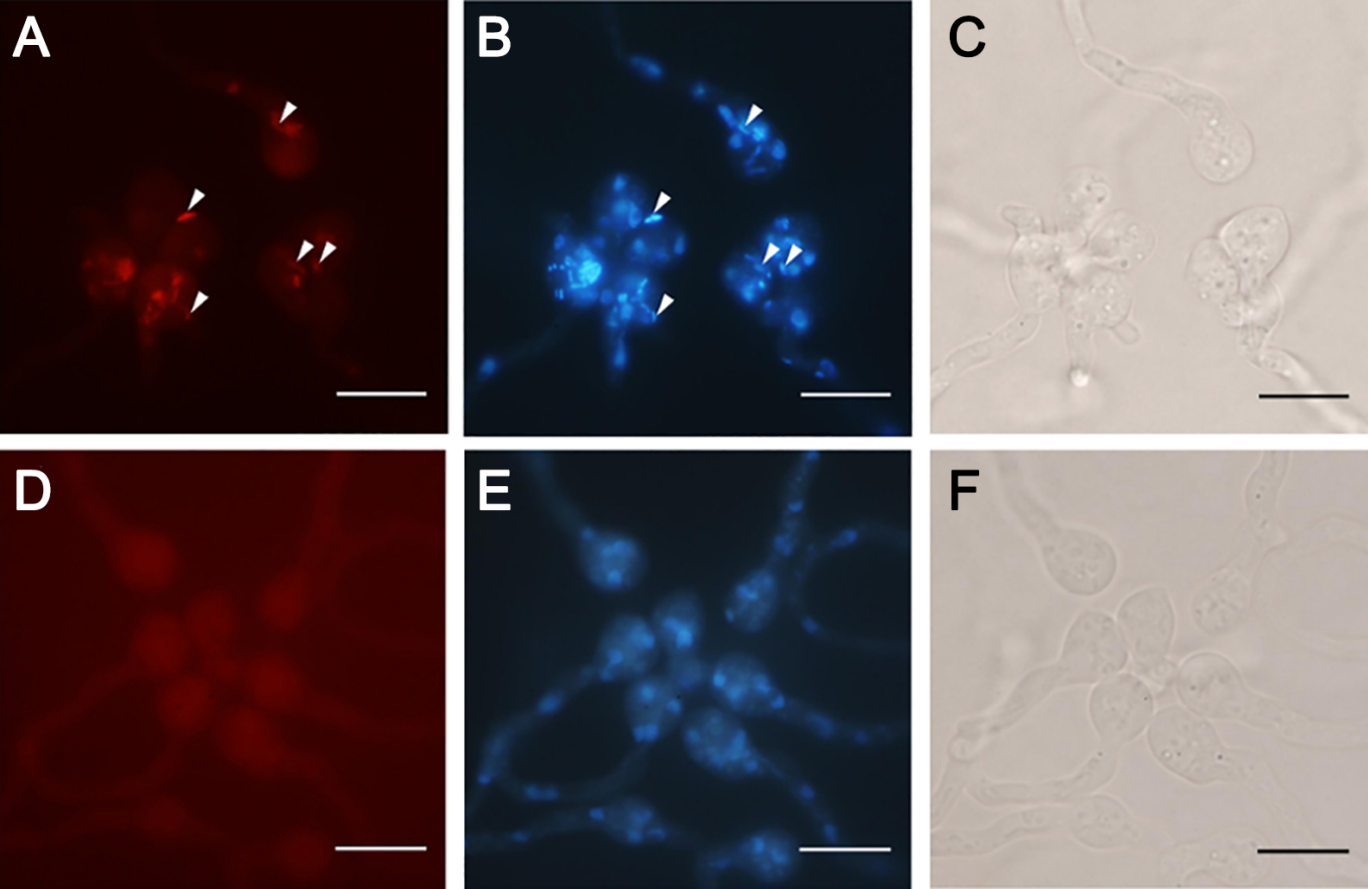
Fluorescence and bright field images of sporangiospores of clonal lines of *Mortierella sugadairana* YTM39 with PCR-positive (B+) and -negative (B–) amplification in diagnostic PCR of the endofungal bacterium. Columns: left; FISH with a Cy3-labeled EUB338 probe (Red), middle; DAPI (blue), right; bright field. Rows: A–C; YTM39s3, D–F; YTM39s14. Rod-shaped endofungal bacterial cells were detected within the sporangiospores of the clonal line YTM39s3 (B+: A, B) by FISH and DAPI staining (arrowheads), and were not detected within YTM39s14 (B–: D, E). Scale bars: 10 μm.

Previous studies successfully eliminated BRE from the host using an antibiotic treatment within the host mycelia of *R. microsporus* (Partida-Martinez *et al.*, 2007) and *M. elongata* (Uehling *et al.*, 2017). In the present study, we established 24 clonal lines of *M. sugadairana* YTM39 by the ciprofloxacin treatment and PCR amplification of the 16S rRNA gene was not observed in these clonal lines (Table 3). These results demonstrated that the ciprofloxacin treatment was effective not only for eliminating BRE-harboring isolates of *R. microsporus*, but also those of *M. sugadairana*. On the other hand, 97 clonal lines of *M. sugadairana* YTM39 were established by single-sporangiospore isolation and PCR amplification of the 16S rRNA gene was observed in 83.5% (81 out of 97) of clonal lines (Table 4). Since the production of the sporangiospores of clonal lines was not related to the presence of BRE, the presence and absence of the bacterium were confirmed in the sporangiospores of the BRE-harboring clonal line (Fig. 2A, B, and C) and BRE-free clonal line (Fig. 2D, E, and F), respectively, by FISH. Therefore, we obtained 24 and 16 bacteria-free clonal lines by the ciprofloxacin treatment and single-sporangiospore isolation, respectively (Table 3 and 4). This result indicated that BRE associated with *M. sugadairana* YTM39 was eliminated from host fungal cells by the successive subculturing of single spores, similar to *G. margarita* (Bianciotto *et al.*, 2004; Lumini *et al.*, 2007), *M. elongata* (Sato *et al.*, 2010), and *R. microsporus* (Mondo *et al.*, 2017). Our mycelial growth assay showed that the presence/absence of BRE and the ciprofloxacin treatment had no harmful effects on mycelial growth by the fungus (Fig. S1). This result ensured that the observation of zygospore formation in the present study was not affected by mycelial growth differences among clonal lines.

### The bacterial endosymbiont inhibits the sexual development of its fungal host

In the present study, six and sixteen clonal lines of *M. sugadairana* YTM39 were used for zygospore induction, which was achieved by the ciprofloxacin treatment and single-sporangiospore isolation, respectively (Table 2). Among them, 10 out of 16 clonal lines obtained by single-sporangiospore isolation harbored BRE (B+) and the other 12 clonal lines were BRE-free (B–) (Table 2). Two isolates of *M. sugadairana*, YTM128 and SUT-174, were used as representative bacteria-free isolates for zygospore induction. Regarding the results of zygospore induction, fungal colonies with hyphal masses were observed in all clonal lines of *M. sugadairana* YTM128 and SUT-174, and most of the BRE-free (B–) clonal lines of *M. sugadairana* YTM39 (Fig. 1A, B, D, and E). Zygospores were always produced in the hyphal masses (Fig. 1A’ and A”; magnified images of hyphal masses in clonal lines of *M. sugadairana* YTM39s9 [Fig. 1A], and other magnified images of *M. sugadairana* YTM39s3_mc1 [Fig. 1B] and YTM39s12 [Fig. 1C] were shown in Fig. S2). On the other hand, hyphal masses were not observed in the BRE-harboring clonal lines of *M. sugadairana* YTM39 (B+) (Fig. 1G and H), except for the clonal line *M. sugadairana* YTM39s12 (Fig. 1C). Therefore, zygospore formation by the fungus was inhibited by the presence of the bacterium (9 out of 10 clonal lines) (Table 2). These results showed the sexual spore infertility of *M. sugadairana* YTM39 by the presence of the bacterium. Even though an exceptional case was found in the BRE-harboring clonal line *M. sugadairana* YTM39s12 showing trace zygospore formation (Fig. 1C), this result is the first to show that BRE inhibited zygospore formation by its fungal host. It currently remains unclear why exceptional cases occurred in the clonal lines *M. sugadairana* YTM39s1 (B–) and YTM39s12 (B+). However, we presumed that zygospore inhibition was related to a population of the bacterium within the mycelia.

### An evolutionary perspective of the symbiotic status of *M. sugadairana* and BRE

The sexual spore infertility of the fungal host by the presence of BRE in the present study raises a question about the relationship between BRE and their Mucoromycotan hosts. Mondo *et al.* (2017) predicted that the current *R. microsporus*-BRE association originated from an antagonistic relationship. This is because the control of the expression of a *ras2-1* gene, encoding a small GTP-protein responsible for reproductive development was hijacked by BRE in *R. microsporus* (Mondo *et al.*, 2017). If the *M. sugadairana*-BRE association follows a similar evolutionary trajectory to that of the *R. microsporus*-BRE association introduced by Mondo *et al.* (2017), as shown in Supplementary Fig. 4, the *M. sugadairana*-BRE association may represent an “early symbiotic status” among Mucoromycota-BRE associations. Further comparative transcriptome analyses between BRE-harboring and BRE-free clonal lines of *M. sugadairana* YTM39 and among different BRE-harboring fungal species are needed to answer this open question.

## Citation

Takashima, Y., Degawa, Y., Nishizawa, T., Ohta, H., and Narisawa, K. (2020) Aposymbiosis of a *Burkholderiaceae*-Related Endobacterium Impacts on Sexual Reproduction of Its Fungal Host. *Microbes Environ ***35**: ME19167.

https://doi.org/10.1264/jsme2.ME19167

## Supplementary Material

Supplementary Material

## Figures and Tables

**Table 1.** Isolates of *Mortierella sugadairana* used in the present study

Isolate name	Culture collection No.	Endofungal bacterium	Collection site	Isolation source
YTM39	NBRC 112366	+	Rakuno Gakuen University, Ebetsu-shi, Hokkaido, Japan	Root, tomato seedling
YTM128	NBRC 112976	–	Midorigaoka Park, Obihiro-shi, Hokkaido, Japan	Soil under *Abies veitchii*
SUT-174	NBRC 104553	–	Mountain Science Center Sugadaira Research Station, University of Tsukuba, Ueda-shi, Nagano, Japan	Decayed twig, *Fagus crenata*

**Table 2.** Zygospore induction of clonal lines originating from *Mortierella sugadairana* YTM39, YTM128, and SUT-174 obtained by single-sporangiospore isolation and the ciprofloxacin treatment.

Name of clonal lines^a^	PCR amplification^b^	Homothallic zygospore formation^c^
YTM39s3	P	–
YTM39s4	P	–
YTM39s7	P	–
YTM39s11	P	–
YTM39s12	P	trace
YTM39s13	P	–
YTM39s3_s1	P	–
YTM39s3_s2	P	–
YTM39s4_s1	P	–
YTM39s4_s2	P	–
YTM39s1	N	–
YTM39s2	N	+
YTM39s6	N	+
YTM39s9	N	+
YTM39s10	N	+
YTM39s14	N	+
YTM39mc1	N	+
YTM39mc2	N	+
YTM39s3_mc1	N	+
YTM39s3_mc2	N	+
YTM39s4_mc1	N	+
YTM39s4_mc2	N	+
YTM128s1	None	+
YTM128s2	None	+
SUT-174s1	None	+
SUT-174s2	None	+
SUT-174s3	None	+
SUT-174s4	None	+

a: Names including “s” and “mc” indicate clonal lines obtained by single-sporangiospore isolation and the ciprofloxacin treatment, respectively. b: P and N indicate positive and negative PCR amplification in diagnostic PCR, respectively. “None” indicates BRE-free isolates since original isolates were obtained. c: +; zygospore formation in both or either of _LC_A and HSA media, –; zygospores were not formed in _LC_A or HSA media. trace; trace zygospore formation was noted in _LC_A medium.

**Table 3.** Elimination efficiency of the endofungal bacterium from *Mortierella sugadairana* YTM39 by the ciprofloxacin treatment.

Origin	Incubation period for DNA extraction (day)	No. of total clonal lines	No. of PCR positive clonal lines	Elimination efficiency
YTM39	7	4	0	100%
YTM39s3	7	10	0	100%
YTM39s4	7	10	0	100%
Total	—	24	0	100%

**Table 4.** The number of PCR-positive clonal lines of *Mortierella sugadairana* YTM39 obtained by serial single-sporangiospore isolation and the frequency of vertical transmission by the endofungal bacterium through sporangiospores.

Origin	Incubation period for DNA extraction (day)	Series of single-sporangiospore isolation	No. of total clonal lines	No. of PCR-positive clonal lines	Frequency of vertical transmission
YTM39	6, 7, 12*	Wild-type	12	6	50%
YTM39	9	Wild-type	34	34	100%
YTM39s3	7	1^st^	10	9	90%
YTM39s4	7	1^st^	10	10	100%
YTM39s3s1	8	2^nd^	7	7	100%
YTM39s3s1s1	7	3^rd^	8	7	87.5%
YTM39s3s1s6	7	3^rd^	8	0	0%
YTM39s3s1s6	7	3^rd^	8	8	100%
Total	—	—	97	81	83.5%

*: The presence of BRE in twelve clonal lines was checked by diagnostic PCR three times with different incubation periods and the results obtained were identical.
