# Supplementary material for: Aposymbiosis of a Burkholderiaceae-Related Endobacterium Impacts on Sexual Reproduction of Its Fungal Host
Source: Microbes Environ. 2020 Apr 15;35(2):ME19167. doi: 10.1264/jsme2.ME19167 (PMC7308579; doi:10.1264/jsme2.ME19167)

Microbes and Environments

Supplementary data

Title:

Aposymbiosis of a *Burkholderiaceae*-related endobacterium impacts on sexual reproduction of its fungal host

Authors and affiliations: Yusuke Takashima[1, 2], Yousuke Degawa[3], Tomoyasu Nishizawa[1, 2], Hiroyuki Ohta[1, 2], Kazuhiko Narisawa* [1, 2]

[1] United Graduate School of Agricultural Science, Tokyo University of Agriculture and Technology, 3-5-8 Saiwai-cho, Fuchu-shi, Tokyo 183-8509, Japan

[2] Ibaraki University College of Agriculture, 3-21-1 Chuo, Ami-machi, Ibaraki, 300-0393, Japan

[3] Sugadaira Research Station Mountain Science Center, University of Tsukuba, 1278-294, Sugadaira, Nagano 386-2204, Japan

Topic of the manuscript: microbial interactions and interrelations with other organisms

Running headline: Zygospore infertility by endobacterium

***Corresponding author:**

Kazuhiko Narisawa

Tel: +81 29-888-8667

Fax: +81 29-888-8667

Email: kazuhiko.narisawa.kkm@vc.ibaraki.ac.jp

E-mail address of other authors:

Yusuke Takashima: yusuke.takashima.senmu@gmail.com

Yousuke Degawa: degawa@sugadaira.tsukuba.ac.jp

Tomoyasu Nishizawa: tomoyasu.nishizawa.agr@vc.ibaraki.ac.jp

Hiroyuki Ohta: hiroyuki.ohta.1494@vc.ibaraki.ac.jp

Fig. S1 Diametrical colony growth (mm/day) of clonal lines of *Mortierella sugadairana* YTM39 incubated on malt extract agar with three replicates at 10, 20, and 30 °C. The name surrounded by red frame indicates BRE-free clonal lines. Colony growth with same alphabetical letters on the plot indicates no significant differences (Tukey HSD test, $p<0.01$).

Fig. S2 Appearance of colonies on $_{\mathrm{LC}}$A incubated for 3 weeks at 18 °C for homothallic zygospores induction using clonal lines of *Mortierella sugadairana* (A, A', A'': YTM39s9; B, B', B'': YTM39s3_mc1; C, C', C'': YTM39s12) A, B, C: Hyphal masses appeared at the edge of 90 mm diam petri dishes (Arrows). A', B', C': Magnified images of the section indicated by arrows. A'', B'', C'': Zygospores formed in hyphal masses indicated by arrowheads. Scale bars: A', B', C' 5 mm; A'', B'', C'' 200 µm.

Figure S1

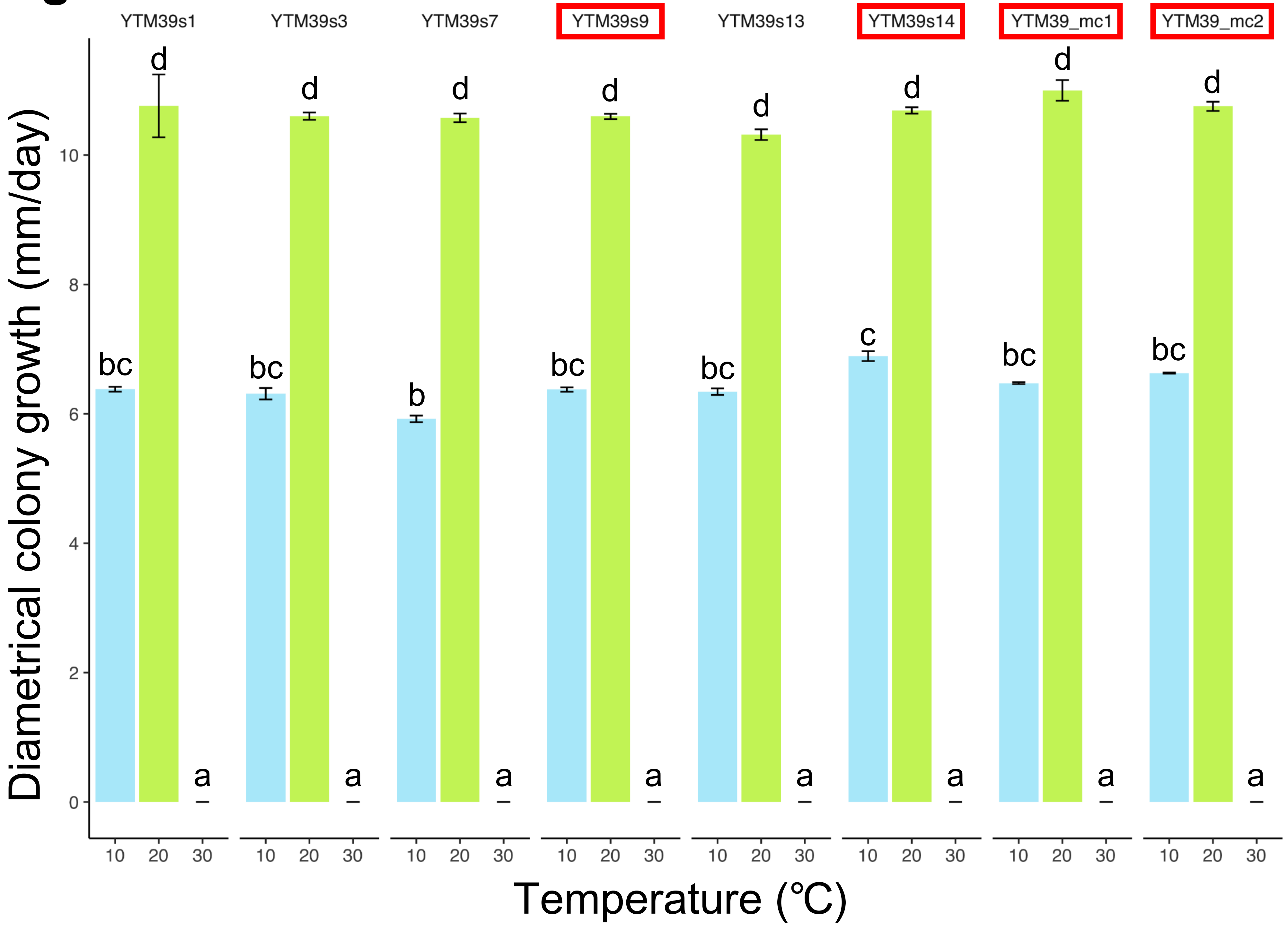

YTM39s1
YTM39s3
YTM39s7
YTM39s9
YTM39s13
YTM39s14
YTM39_mc1
YTM39_mc2
Diametrical colony growth (mm/day)
Temperature (℃)
0
2
4
6
8
10
10 20 30
bc
b
c
d
a

Figure S2

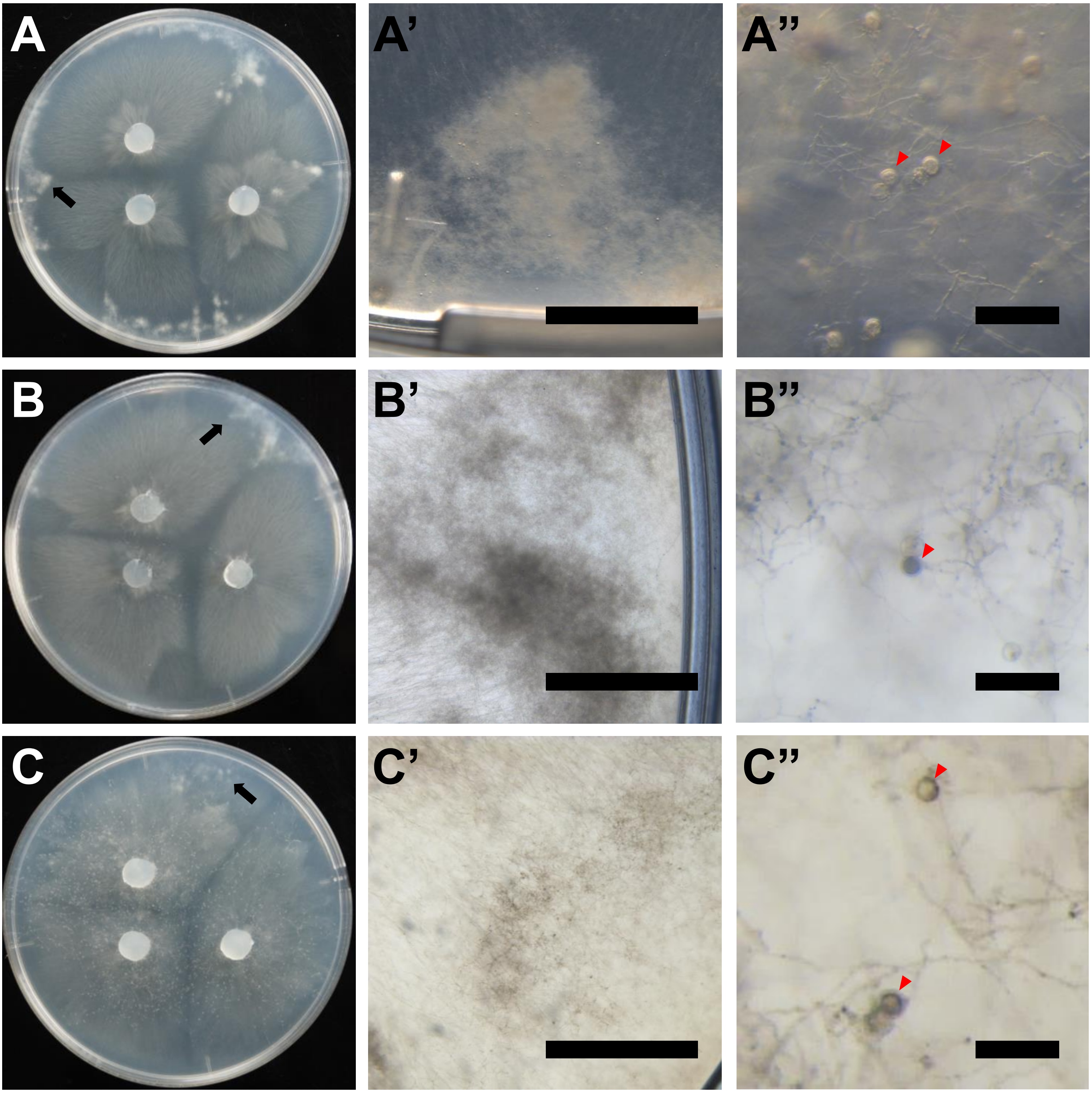

Supplement: Supplementary file 1 — Supplementary Material [file 35_19167_s1.pdf]
